# Fatty Acid Profiling in Kernels Coupled with Chemometric Analyses as a Feasible Strategy for the Discrimination of Different Walnuts

**DOI:** 10.3390/foods11040500

**Published:** 2022-02-09

**Authors:** Qiao Pei, Yongxiang Liu, Shaobing Peng

**Affiliations:** College of Forestry, Northwest A & F University, Yangling 712100, China; peiqiao@nwafu.edu.cn (Q.P.); lyx2020@nwafu.edu.cn (Y.L.)

**Keywords:** fatty acid profiling, geographic variation, probability grading, chemometrics

## Abstract

Walnuts have high oil content in their kernels, and they have attracted considerable attention in the food, beverage, nutrient, and health fields because of their delicious taste and potential health benefits. Fatty acid profiles of kernels vary depending on walnuts species, ontogenic variations, and planting environments. To determine the key indicators that can be used to distinguish different walnuts using chemometric analyses, the fatty acid compositions and contents of 72 walnut samples were measured and evaluated. Three fatty acids, oleic acid (21.66%), linoleic acid (56.40%), and linolenic acid (10.50%), were the most common fatty acid components in the kernels. Palmitic acid and linolenic acid in kernels were found to be indicators to rank the walnuts into five levels. Three groups were identified based on of several chemometrics. Oleic acid in kernels was typical fatty acid that could be used to distinguish three walnut groups based on the results of discriminant analysis, while oleic acid and linoleic acid were key differential fatty acids on the discrimination of each group based on the result of orthogonal partial least squares discriminant analysis. This study provides information on how to classify walnuts from different geographical locations based on kernel fatty acid profiling and provides an approach to identify possible adulterations in walnuts on the markets. Moreover, the results are potentially relevant to quality assessments of walnuts.

## 1. Introduction

*Juglans regia* L., commonly known as Persian walnut or English walnut, is an important woody oil plant due to the high oil content in its kernels with huge economic gains [1,2]. The trees are cultivated in wide regions across the world for their strong adaptation to a variety of climate and soil conditions [3,4], and they play a vital role in conserving soil and water [5]. The edible walnut kernels consist of the embryo (meat) and a seed coat or pellicle [6], and they are rich in a variety of nutrient and bioactive constituents, including proteins [7], lipids [8], phenolic substances [9], phytosterols and squalene [10,11], and various fatty acids [12], and they demonstrate various therapeutic effects, such antioxidant [12], antimicrobial [13], antinociceptive, and anti-inflammatory potentials [11]. Therefore, the kernels have attracted considerable attention in the food, pharmacological, and industrial fields because of these nutrients and potential health benefits. Generally, genotype, cultivated environment, and their interaction between genotype and the environment have affected the accumulation of metabolites in walnut kernels [1,10,12]. Moreover, disruption of the integrity of the seed coat pellicle during shelling operations triggers postharvest deterioration, and dry treatments including roasting have undesirable effects on the flavour, colour, fatty acid profile and bioactive components of kernel [2,6]. Thus, an effective measure to evaluate quality of kernel should be proposed.

Distinguishing walnuts is difficult due to the wide area of cultivation, similarity in appearance of shells, and diverse cultivars and species. Furthermore, walnut products, such as walnut oils, are more difficult to authenticate in terms of species and origin. Since good-quality walnut products are expensive and difficult to identify, adulteration can occur due to misidentifying origins and varieties of walnuts, as well as intentional substitution with inexpensive walnuts, or other fraudulent conduct [6]. Fraudulent behaviour restricts the steady development of high-quality walnuts and damage the interests of consumers. Molecular methods are used to identify adulterants because they are accurate, effective, and not dependant on age, environmental factors, and storage and processing conditions [14,15]. However, extracting DNA or RNA is difficult because of the strict requirements for sampling methods and sample storing. Physical and chemical analyses coupled with chemometrics can be used to detect adulterations in food commodities [16,17]. Therefore, since fatty acids are one of the basic components required by organisms to survive, they can be used instead of molecular methods to distinguish species and origins of food [18,19]. Thus, fatty acid profiling coupled with chemometrics is feasible to authenticate walnuts on the market.

In this study, fatty acid profiling of kernels from 72 walnut samples for different geographic origins and merchants was determined using gas chromatograph mass spectrometry (GC-MS). Then, several chemometric analyses based on fatty acid profiling were used to classify different walnuts and determine the key differential fatty acids between different walnut groups. This study identifies fatty acid variations among kernels from different origins and determines the key fatty acids in kernels that could be used to distinguish walnuts. Moreover, the results are potentially relevant to quality assessments of walnuts.

## 2. Materials and Methods

### 2.1. Sample Collection and Preparation

In 2020, 72 walnut samples were collected from 8 provinces across China (Hebei, Shaanxi, Shandong, Xinjiang, Guizhou, Shanxi, Sichuan, and Yunnan province), which are the main walnut-producing regions. Thirty-three samples were obtained from network providers (W1-W33), and 39 samples were obtained from experimental fields (W34-W72) (Table S1). The varieties and origins of the samples from the experimental fields were known, and the sampling trees were in their full productive period (8–12 years old). Thirty walnut samples were collected at random from each experimental field and then mixed; in addition, 30 walnut samples were obtained from each network provider. Samples from each source were separated into 3 replicates at random (10 walnuts in each replicate) and detailed information was recorded. Then, all samples were sealed in valve bags and transported to the laboratory. The walnut kernels were separated from peels and shells, and then they were each ground into a homogenized powder for fatty acid extraction.

### 2.2. Fatty Acid Extraction and Analysis

**Figure 1:**
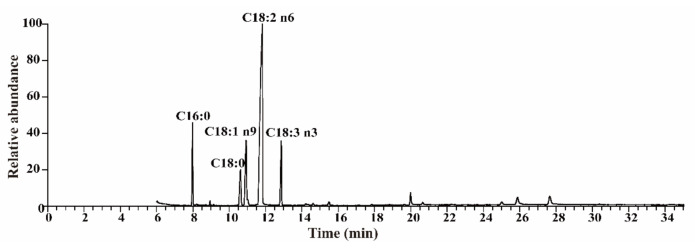
The relative abundance of each fatty acid in the walnut kernel. C16:0, palmitic acid; C18:0, stearic acid; C18:1 n9, oleic acid; C18:2 n6, linoleic acid; C18:3 n3, linolenic acid.

A 3 mL amount of mixture solvent (2:1 chloroform and methanol) and 0.1 g walnut kernel powder were used to extract crude fats in the kernels of walnuts [20], and the extraction was repeated three times for each sample. Then, fatty acid methyl esterification was performed using a mixture solvent (99% methanol and 1% sulfuric acid). Fatty acid methyl esters were dissolved using chloroform and were detected using GC-MS (Thermo Fisher Scientific Inc., Waltham, MA, USA) according to the method outlined in Ma et al. [18]. The amount of each fatty acid was described as a proportion of total fatty acids (%) of the walnut kernel (Figure 1).

### 2.3. Data Analyses

The evaluation of the normally distributed population for the fatty acid data was performed in IBM SPSS Statistics 20.0 software. The cluster heat map (CHM), correlation analysis, principal component analysis (PCA), and discriminant analysis (DA) were conducted using OriginPro 2021 (Originlab, Northampton, MA, USA) based on the mean standardized value for each fatty acid in kernels of walnuts. Orthogonal partial least squares discriminant analysis (OPLS-DA), non-metric multidimensional scaling (NMDS), and uniform manifold approximation and projection for dimension reduction (UMAP) were performed based on the mean standardized value for each fatty acid in kernels of walnuts using the website (https://www.omicshare.com/tools/Home/Soft/getsoft accessed on 10 January 2022).

## 3. Results

### 3.1. Fatty Acid Profiling in Kernels and Probability Grading of Walnuts

**Figure 2:**
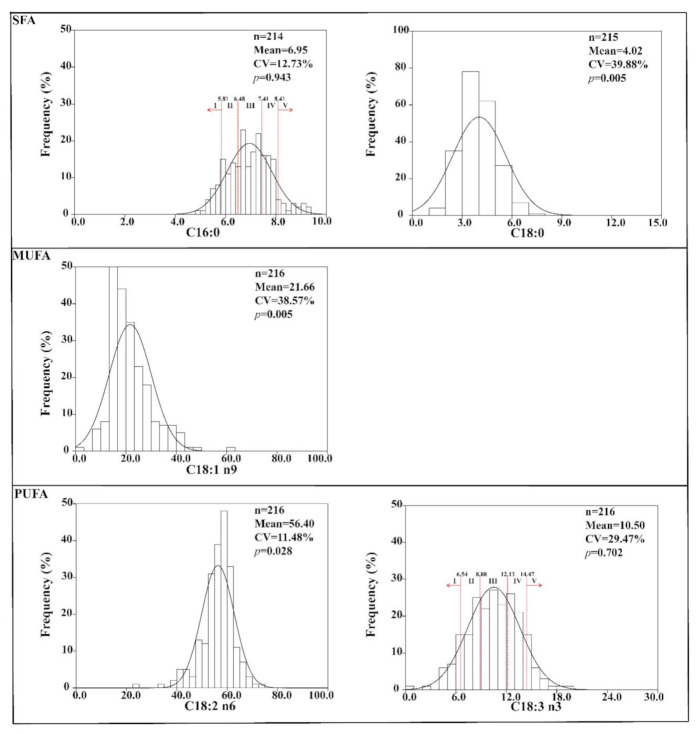
Fatty acid profiling in kernels and probability grading of walnuts. n, amount of valid data; CV, coefficient of variance; *p*, value for the Kolmogorov–Smirnov normality test; C16:0, palmitic acid; C18:0, stearic acid; C18:1 n9, oleic acid; C18:2 n6, linoleic acid; C18:3 n3, linolenic acid; SFA, saturated fatty acids; MUFA, monounsaturated fatty acids; PUFA, polyunsaturated fatty acids.

Fatty acids in kernels of walnuts from different sources varied in contents and components, a total of five dominating fatty acids were detected in the kernel samples (Figure 2). The kernels of walnuts possessed two saturated fatty acids-palmitic acid (C16:0) and stearic acid (C18:0), one monounsaturated fatty acid (oleic acid, C18:1 n9), and two polyunsaturated fatty acids—linoleic acid (C18:2 n6) and linolenic acid (C18:3 n3). C18:1 n9 (21.66%), C18:2 n6 (54.40%), and C18:3 n3 (10.50%) were the most common fatty acid compounds in the kernels of walnuts. The variance coefficients of these fatty acids ranged from 11.48% to 39.88%. The content of C18:2 n6, which had the lowest variance coefficient, ranged from 24.50% to 73.99%, while C18:0, which had the highest variance coefficient, ranged from 1.14% to 21.29%. The lower variance coefficient indicated a good stability of the fatty acid compound from different sources.

The evaluation of the normally distributed population for the fatty acid data was performed by consideration of the *p* value for the Kolmogorov–Smirnov normality test. When the *p* value for the Kolmogorov–Smirnov normality test was more than 0.05, the population distribution of the fatty acid data was normal. The distributions of C16:0 and C18:3 n3 obeyed normal distributions (*p* > 0.05), but the distributions of the remaining fatty acids were abnormal. Based on the fatty acids which obeyed normal distributions, five levels of probability of occurrence (I—10%, II—20%, III—40%, IV—20%, and V—10%) were determined for the threshold values of C16:0 (5.81%, 6.48%, 7.41%, and 8.08%) and C18:3 n3 (6.54%, 8.88%, 12.13%, and 14.47%).

### 3.2. Chemometric Analyses for Walnuts Based on Kernel Fatty Acid Data

To better understand fatty acid differences among different walnut samples, several chemometric methods were used to classify walnut samples and determine the key differences among kernels from different walnut samples.

#### 3.2.1. Cluster Heat Map (CHM)

**Figure 3:**
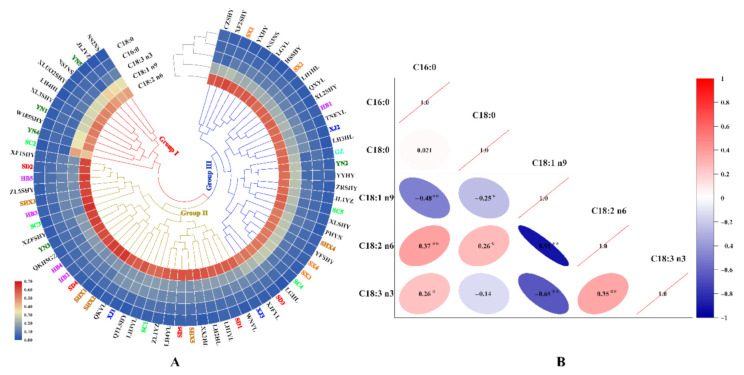
Cluster heat map of different walnuts (**A**) and correlation analysis between each fatty acid (**B**). Codes with the same colour represent samples that belong to the same producing area, and codes with black colour represent the known species; * represents medium correlation (*p <* 0.05); ** represents strong correlation (*p <* 0.01); C16:0, palmitic acid; C18:0, stearic acid; C18:1 n9, oleic acid; C18:2 n6, linoleic acid; C18:3 n3, linolenic acid.

First, CHM, an unsupervised pattern recognition method, was used to classify the 72 walnuts and cluster similar fatty acids based on Euclidean distance with the complete cluster method (Figure 3A). The differences in fatty acid content of kernels from each original sample were also observed. C16:0 and C18:0 were present with similar content trends in the kernels, while the remaining fatty acids were different. Moreover, correlation analysis was demonstrated based on fatty acid data (Figure 3B). C16:0 was significantly negatively correlated with C18:1 n9 and significantly positively correlated with C18:2 n6 (*p* < 0.01), while C16:0 was positively correlated with C18:2 n6 (*p* < 0.05). C18:0 was negatively correlated with C18:1 n9 and positively correlated with C18:2 n6 (*p* < 0.05). C18:1 n9 was significantly negatively correlated with C18:2 n6 and C18:3 n3 (*p* < 0.01). C18:2 n6 was significantly positively correlated with C18:3 n3 (*p* < 0.01). The samples were separated into three dispersed groups. Some samples from different species or origins were gathered together, while some samples from same species or origin were separated, illustrating that the accumulation of fatty acids in kernels was complicated, and affected by genotype, cultivated environment, and the interaction between genotype and the environment.

#### 3.2.2. Principal Component Analysis (PCA)

**Figure 4:**
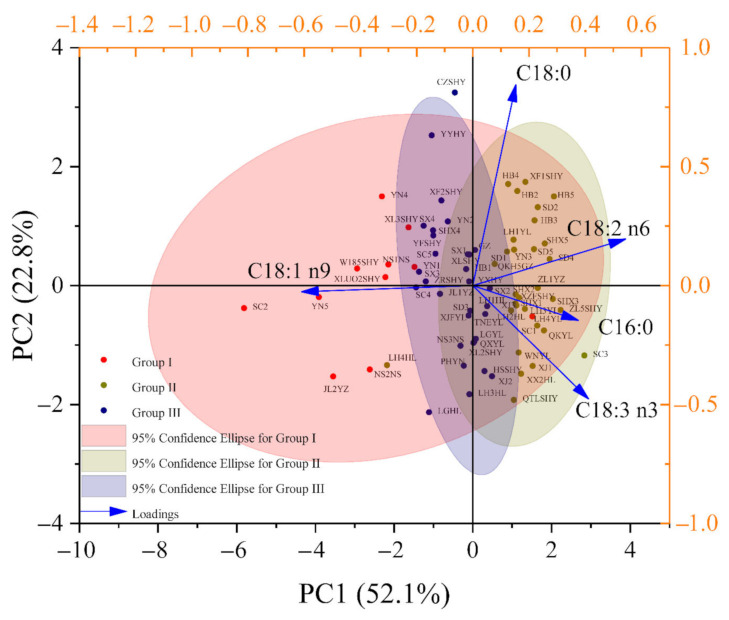
Principal component analysis based on fatty acid data. Sample spots with the same colour represent the samples that belong to the same group. PC, principal component; C16:0, palmitic acid; C18:0, stearic acid; C18:1 n9, oleic acid; C18:2 n6, linoleic acid; C18:3 n3, linolenic acid.

PCA was used to better understand the distribution characteristics of samples (Figure 4). Two PCs whose eigenvalue roots were greater than one were generated from the original data, accounting for 74.9% of the variation (PC1—52.1% and PC2—22.8%), with only a 25.1% loss of information. Among five fatty acids contributed to two principal components, C18:0, C18:1 n9, and C18:2 n6 were far from the origin of coordinates and contributed more to the variation of each axis. C18:1 n9 with high negative loadings and C18:2 n6 with high positive loadings contributed more to the variation of PC1, while C18:0 with high positive loadings contributed more to the variation of PC2. Thus, these highly contributing fatty acids could be used to distinguish walnuts from different groups, and they may be the key components in evaluating walnut quality. Among these walnut samples, LH4YL was gathered in the outside of Group I, LH4HL was gathered in the outside of Group II, and CZSHY was gathered in the outside of Group III. Thus, this discrepancy in fatty acid compositions among kernels from these walnuts should be explored.

#### 3.2.3. Non-Metric Multi-Dimensional Scaling (NMDS)

**Figure 5:**
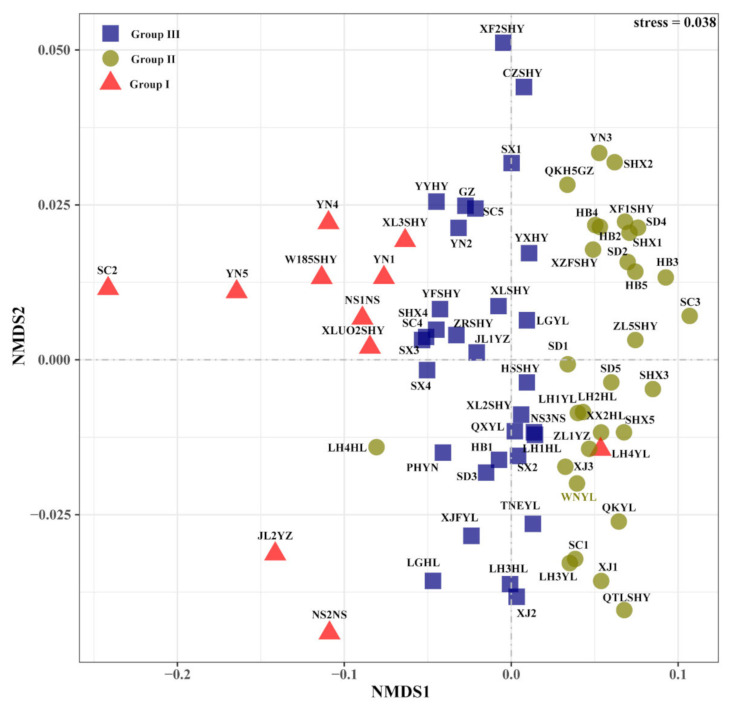
Non-metric multi-dimensional scaling based on fatty acid data. Sample spots with same colour and symbol represent the samples that belong to the same group; NMDS, non-metric multi-dimensional scaling.

Unlike CHM and PCA, NMDS is a nonlinear model and better reflects the nonlinear structure of the data in Figure 5. The stress of the model based on Bray–Curtis distance was 0.038, indicating the goodness of fit was sufficient. Among these walnut samples, the sample (LH4YL) was gathered in the outside of Group I, while LH4HL was gathered in the outside of Group II.

#### 3.2.4. Uniform Manifold Approximation and Projection for Dimension Reduction (UMAP)

**Figure 6:**
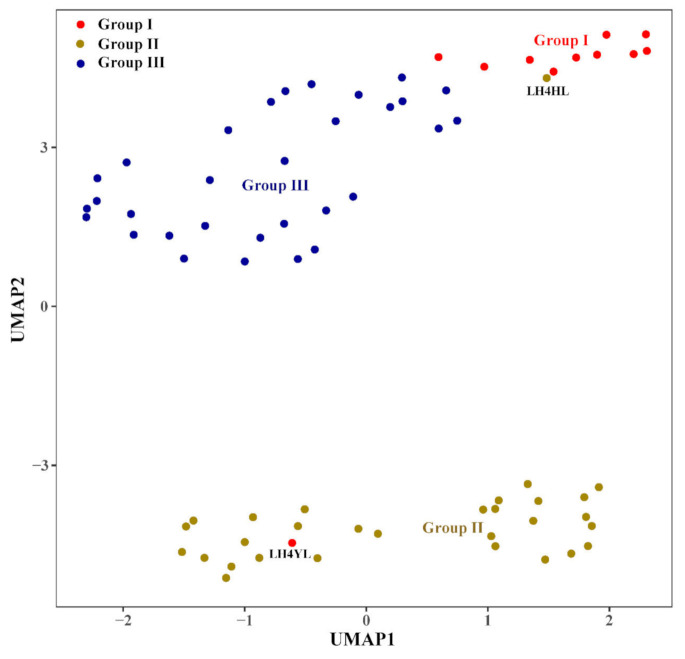
Uniform Manifold Approximation and Projection for Dimension Reduction based on fatty acid data. Sample spots with same colour represent the samples that belong to the same group. UMAP, Uniform manifold approximation and projection for dimension reduction.

Another nonlinear model, UMAP tended to better retain the global structure of data and visualized the distribution of walnut samples (Figure 6). Three discriminant groups (Group I, Group II, and Group III) were generated. The spots in the figure represent the distribution of walnut samples, reflecting the similarity between the samples. The closer the spots, the greater the similarity of the fatty acid profile in these samples. Among these walnut samples, LH4YL was gathered in the outside of Group I, while LH4HL was gathered in the outside of Group II.

#### 3.2.5. Discriminant Analysis (DA)

**Figure 7:**
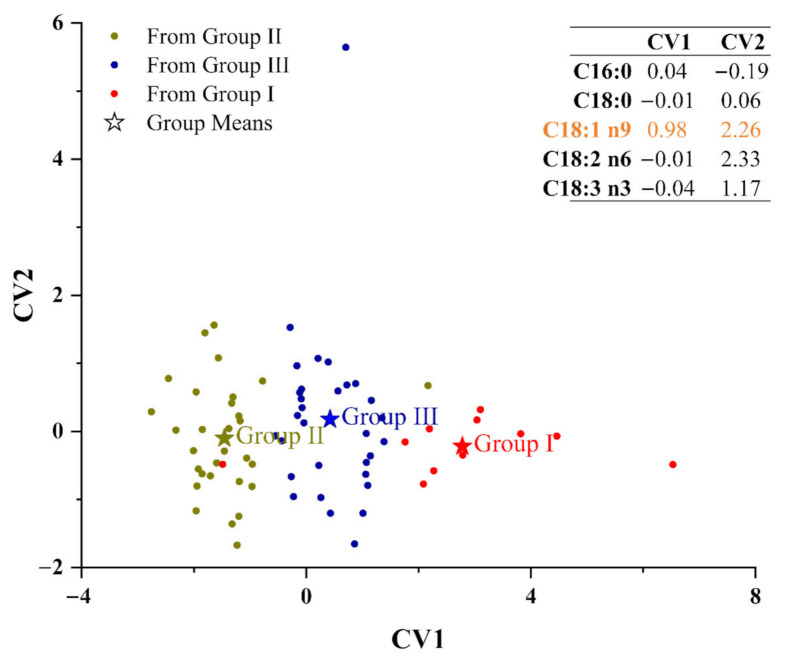
Discriminant analysis based on fatty acid data. Sample spots with same colour represent the samples that belong to the same group; CV, canonical variable; C16:0, palmitic acid; C18:0, stearic acid; C18:1 n9, oleic acid; C18:2 n6, linoleic acid; C18:3 n3, linolenic acid.

DA, as a supervised analysis technique, was used to better understand the categorization of the 72 samples (Figure 7). Three distinct groups (Group I, Group II, and Group III) were generated. For the discrimination function, C18:1 n9 in the kernels was a typical fatty acid that was used to distinguish three walnut groups. The standard of discrimination was as follows: five fatty acids were substituted into two equations, and then the unknown samples were assigned to a group by compared to the value means of the canonical variable (CV) of the training group date, CV1 (2.78 for Group I, −1.46 for Group II, and 0.42 for Group III) and CV2 (−0.22 for Group I, −0.10 for Group II, and 0.18 for Group III). Cross-validation proved 5.22% of the error rate (9.09% for Group I, 3.33% for Group II, and 3.23% for Group III), indicating that the discrimination of samples from Group I, Group II, and Group III could generate a little error. If LH4YL was classified into the Group II and LH4HL was in Group I, cross-validation proved 3.03% of the error rate (9.09% for Group I, 0.00% for Group II, and 0.00% for Group III). Thus, LH4YL should be classified into Group II and LH4HL into Group I.

#### 3.2.6. Orthogonal Partial Least Squares Discriminant Analysis (OPLS-DA)

Since fatty acid constituents in the kernels varied in different groups (Group I, Group II, and Group III), OPLS-DA was applied to determine the important differential fatty acid to distinguish each group (Table 1). The predictive abilities of Group I vs. II and Group II vs. III were better, with the explanatory rates being more than 0.5 based on R2X, R2Y, and Q2Y, while the predictive ability of Group I vs. III was acceptable, with the explanatory rates being more than 0.4 based on R2Y and Q2Y. The influence of intensity and the explanatory ability of each indicator on the discrimination of each group can be measured via the values of variables important in projection (VIP). The indicators included in the model were important when the value of VIP was no less than one. The VIP values varied in distinguishing kernels from different groups. The VIP values of C18:1 n9 and C18:2 n6 were more than one in distinguishing samples of Group I and Group II, Group I and Group III, and Group II and Group III. Thus, C18:1 n9 and C18:2 n6 were key differential fatty acids on the discrimination of each group.

### 3.3. Fatty Acid Difference in Kernels among Different Groups

**Figure 8:**
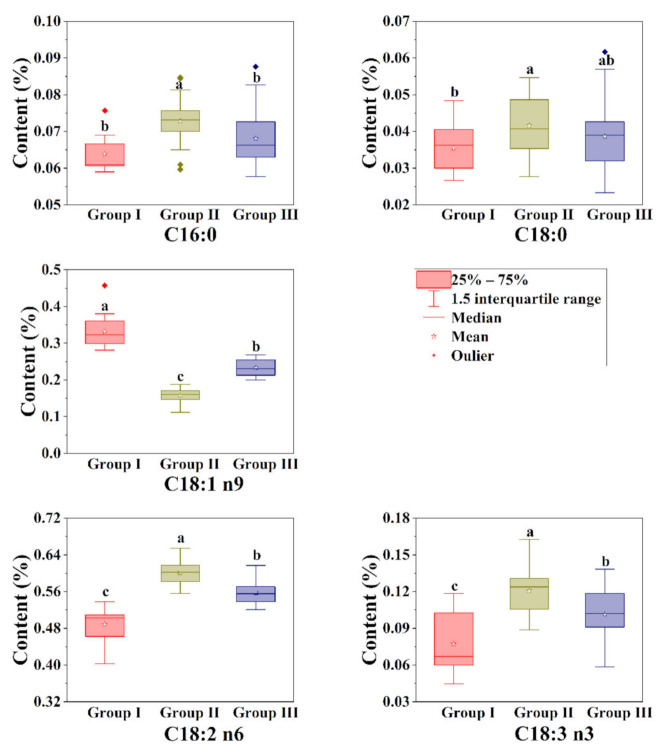
The fatty acid profile in kernels among different groups. The different letters (a, b, c, or ab) behind the results indicate significant differences among different groups (*p* < 0.05). C16:0, palmitic acid; C18:0, stearic acid; C18:1 n9, oleic acid; C18:2 n6, linoleic acid; C18:3 n3, linolenic acid.

Based on the results of probability grading and chemometric analyses, all fatty acids play important roles in distinguishing samples. The fatty acid differences in kernels among the groups are shown in Figure 8. All fatty acids were significantly different among different groups (*p* < 0.05), and the differences in content of C16:0 between Group I and Group III was not significant. The kernels of Group II possessed the highest contents in C16:0, C18:0, C18:2 n6 and C18:3 n3, while it possessed the highest contents in C18:1 n9. In consideration of key fatty acids (C18:1 n9 and C18:2 n6), the kernels of walnuts in Group I demonstrated high C18:1 n9 and low C18:2 n6, the kernels of walnuts in Group II demonstrated low C18:1 n9 and high C18:2 n6, and the kernels of walnuts in Group III demonstrated moderate C18:1 n9 and C18:2 n6.

## 4. Discussion

Fatty acid profiling is a good indicator for evaluating the quality and stability of oils [21]. Kernels are staple products from walnut trees, and they are widely used for food, oil extraction, and as raw materials for the processing industry [7,13]. Thus, determining fatty acid profiling in walnut kernels is necessary. The fatty acids in organisms are easily affected by organism genotypes [22], growth environment [23], or a combination of genotypes and environments [18,24]. Walnut species and environments in which the species are cultivated were the main factors that caused fatty acid variations in kernels from different network providers and experimental fields. C18:1 n9, C18:2 n6, and C18:3 n3 are the dominating fatty acids in the kernels of walnuts, and they are also present in high amounts at different developed stages [25]. Moreover, the dominating contents of C18:1 n9, C18:2 n6, and C18:3 n3 in kernel have also been found in Iranian walnuts [26]. The kernels of walnuts are often used for food due to their considerable nutritional and functional values. Due to the presence of various functional fatty acids (monounsaturated fatty acid and polyunsaturated fatty acids), walnut products are beneficial for human health. Many human health issues are related to the ratio of ω-6/ω-3, which act in combination to regulate several physiological processes as part of human diets [27,28]. The ratio of ω-6/ω-3 in fatty acids is usually used to analyse the quality of nutritional oil and fat, and values lower than 4.0 are recommended by the UK Department of Health [29]. The values of ω-6/ω-3 ranged from 2.68 to 12.59, indicating that some walnuts should not be consumed regularly. The C16:0 and C18:3 n3 obeyed normal distributions, and they were used to group walnuts into five levels (I—10%, II—20%, III—40%, IV—20%, and V—10%) on the basis of their threshold values. The levels and indicators were different from the four levels of the Chinese standard for walnut quality (GB/T 20398-2006) based on sensory, physical, and chemical indicators [30]. The indicators of the Chinese standard for walnut quality are simple to use to group walnuts, but they are easily affected by processing and storage methods. The fatty acids used in this study could eliminate additional influence factors and group walnuts more realistically. The probability grouping based on fatty acid proportions should be a supplement for grouping walnut qualities.

*J. regia* and some other species in the genera *Juglans* and *Carya* are botanically related, and they are often confused due to their similarities in terms of morphological characteristics, especially in terms of the shells and kernels of walnuts. However, some obvious variations in physio-biochemical characteristics were found in samples from different plantations and species1. Fatty acid profiling coupled with chemometrics highlights the differentiation of samples obtained from different species and different origins. Six chemometric methods (CHM, PCA, NMDS, UMAP, DA, and OPLS-DA) were used to classify walnut origins and determine the key differences among kernels from different geographic origins based on the mean normalized values of fatty acids. First, the walnut samples from 72 origins were separated into three groups (Group I, Group II, and Group III). Although NMDS, UMAP, HCA, and PCA were all unsupervised, HCA and PCA were linear models, but NMDS and UMAP were nonlinear models [17,31,32]. The classification results of NMDS, UMAP, and PCA were different from those of HCA due to the diverse operational forms. C18:1 n9 in kernels was the typical fatty acid that could be used to distinguish three walnut groups based on the results of DA, while C18:1 n9 and C18:2 n6 were key differential fatty acids in the discrimination of each group based on the results of OPLS-DA. Higher frequency of C18:1 n9 and C18:2 n6 has also been found when discriminating Iranian walnuts, indicating the effectiveness of these fatty acids in discriminating samples [26].

In overall consideration of chemometric classification, LH4YL was classified into Group II and LH4HL into Group I. The fatty acid differences in kernels across all groups were diverse. Genotype and environment were the main factors that affected plants, which responded to environmental changes under different biotic and abiotic stresses [24]. The geographical origins in our study remarkably influenced fatty acid content and composition in kernels. The fatty acid variations caused by geographical origins were also found in Iranian walnuts from six regions [26] and other species [18]. The fatty acids in the same group from different origins varied significantly, and the amounts of fatty acids in walnuts from some origins were abnormal. The influence of environmental factors on the fatty acid composition in different species was diverse, and location, climate, and soils were the main issues [18]. Thus, the detailed relationship between fatty acid profiling and correlated influence factors should be explored in future research.

## 5. Conclusions

In this study, five main fatty acids in kernels of walnuts were determined among the 72 samples, and C18:1 n9, C18:2 n6, and C18:3 n3 were the dominating fatty acids in the kernels of walnuts. The geographical origins in our study remarkably influenced the fatty acid content and composition in kernels. The C16:0 and C18:3 n3 obeyed normal distributions, and they were used to group walnuts into five levels (I—10%, II—20%, III—40%, IV—20%, and V—10%). Chemometric analyses highlighted the variations of walnuts from different origins and determined the important fatty acids that could be used to distinguish different walnuts. C18:1 n9 in kernels was a typical fatty acid that could be used to distinguish three walnut groups based on the results of DA, while C18:1 n9 and C18:2 n6 were key differential fatty acids in the discrimination of each group based on the results of OPLS-DA. Furthermore, the results of this study proved it was possible to use fatty acid profiling to determine the major variations in the compositions of different walnuts from different origins. The detailed relationship between fatty acid profiling and correlative influence factors should be explored in future research.

## Figures and Tables

**Table 1:** Variables important in projection and verification by permutation test between different groups.

Fatty Acid	Group I vs. II	Group I vs. III	Group II vs. III
C16:0	0.088	0.073	0.093
C18:0	0.064	0.037	0.042
C18:1 n9	1.828	1.783	1.877
C18:2 n6	1.208	1.260	1.127
C18:3 n3	0.430	0.476	0.443
R^2^X	0.999	0.998	0.998
R^2^Y	0.711	0.486	0.630
Q^2^Y	0.686	0.423	0.596

C16:0, palmitic acid; C18:0, stearic acid; C18:1 n9, oleic acid; C18:2 n6, linoleic acid; C18:3 n3, linolenic acid; R^2^X represents the explanatory rate for the X matrix in the model; R^2^Y represents the explanatory rate for the Y matrix in the model; Q^2^Y represents the predictive ability of the model. In theory, the model is better when R^2^X, R^2^Y, and Q^2^Y are closer to one; usually, the model is better if R^2^X, R^2^Y, and Q^2^Y are higher than 0.5, and it is acceptable if R^2^X, R^2^Y, and Q^2^Y are higher than 0.4.

## Data Availability

Data is contained within the article (or Supplementary Materials).

## Supplementary Materials

The following supporting information can be downloaded at: https://www.mdpi.com/article/10.3390/foods11040500/s1, Table S1. The detailed information on sample sources of different walnuts.

## Abbreviations

C16:0, palmitic acid; C18:0, stearic acid; C18:1 n9, oleic acid; C18:2 n6, linoleic acid; C18:3 n3, linolenic acid; CV, coefficient of variance; SFA, saturated fatty acids; MUFA, monounsaturated fatty acids; PUFA, polyunsaturated fatty acids; CHM, cluster heat map; PCA, principal component analysis; DA, discriminant analysis; OPLS-DA, orthogonal partial least squares discriminant analysis; NMDS, non-metric multidimensional scaling.
