# Supplementary material for: Fatty Acid Profiling in Kernels Coupled with Chemometric Analyses as a Feasible Strategy for the Discrimination of Different Walnuts
Source: Foods. 2022 Feb 9;11(4):500. doi: 10.3390/foods11040500 (PMC8871327; doi:10.3390/foods11040500)
Supplement: Supplementary file 1 [file foods-11-00500-s001.zip › foods-1580271-supplementary.pdf]

Supplementary tables

Fatty Acid Profiling in Kernels Coupled with Chemometric Analyses as a Feasible Strategy for the Discrimination of Different Walnuts

Qiao Pei, Yongxiang Liu, and Shaobing Peng *

College of Forestry, Northwest A & F University, Yangling 712100, China; peiqiao@nwafu.edu.cn (Q.P.); lyx2020@ nwafu.edu.cn (Y.L.); pshaobing@nwsuaf.edu.cn (S.P.)

* Correspondence: pshaobing@nwsuaf.edu.cn (S.P.)

Supplementary tables

Table S1 The detailed information on sample sources of different walnuts.

Table S1 **The detailed information on sample sources of different walnuts.**

Code	Name	Province	Country
W1	HB1	Hebei	Qianxi
W2	HB2	Hebei	Zanhuang
W3	HB3	Hebei	Zanhuang
W4	HB4	Hebei	Gaobeidian
W5	HB5	Hebei	Zanhuang
W6	SHX1	Shaanxi	Lintong
W7	SHX2	Shaanxi	Shangzhou
W8	SHX3	Shaanxi	Xian
W9	SHX4	Shaanxi	Fuping
W10	SHX5	Shaanxi	Dali
W11	SD1	Shandong	Feicheng
W12	SD2	Shandong	Zaozhuang
W13	SD3	Shandong	Feicheng
W14	SD4	Shandong	Shanting
W15	SD5	Shandong	Dongping
W16	XJ1	Xinjiang	Akesu
W17	XJ2	Xinjiang	Akesu
W18	XJ3	Xinjiang	Akesu
W19	GZ1	Guizhou	Liuzhi
W20	SX2	Shanxi	Hongtong
W21	SX3	Shanxi	Jiangxian
W22	SX4	Shanxi	Xiaoyi
W23	SX5	Shanxi	Yancheng
W24	SC1	Sichuan	Xindu
W25	SC2	Sichuan	Renhe
W26	SC3	Sichuan	Pengzhou
W27	SC4	Sichuan	Yanyuan
W28	SC5	Sichuan	Xindu
W29	YN1	Yunnan	Yuxi
W30	YN2	Yunnan	Zhanyi
W31	YN3	Yunnan	Dali
W32	YN4	Yunnan	Dali
W33	YN5	Yunnan	Dongchuan
W34	LH1HL	Shaanxi	Huanglong
W35	LH2HL	Shaanxi	Huanglong
W36	LH3HL	Shaanxi	Huanglong
W37	LH4HL	Shaanxi	Huanglong
W38	LGHL	Shaanxi	Huanglong
W39	XX2HL	Shaanxi	Huanglong

W40	LH1YL	Shaanxi	Yangling
W41	LH3YL	Shaanxi	Yangling
W42	LH4YL	Shaanxi	Yangling
W43	LGYL	Shaanxi	Yangling
W44	QXYL	Shaanxi	Yangling
W45	TNEYL	Shaanxi	Yangling
W46	WNYL	Shaanxi	Yangling
W47	QKYL	Shaanxi	Yangling
W48	XJFYL	Shaanxi	Yangling
W49	CZSHY	Shaanxi	Shanyang
W50	XLUO2SHY	Shaanxi	Shanyang
W51	XLSHY	Shaanxi	Shanyang
W52	QTLSHY	Shaanxi	Shanyang
W53	XL2SHY	Shaanxi	Shanyang
W54	XL3SHY	Shaanxi	Shanyang
W55	ZL5SHY	Shaanxi	Shanyang
W56	W185SHY	Shaanxi	Shanyang
W57	XF1SHY	Shaanxi	Shanyang
W58	XF2SHY	Shaanxi	Shanyang
W59	ZRSHY	Shaanxi	Shanyang
W60	YFSHY	Shaanxi	Shanyang
W61	XZFSHY	Shaanxi	Shanyang
W62	HSSHY	Sichuan	Hanyuan
W63	YYHY	Sichuan	Hanyuan
W64	YXHY	Sichuan	Hanyuan
W65	QKH5GZ	Guizhou	Nanming
W66	JL1YZ	Shanxi	Yingze
W67	JL2YZ	Shanxi	Yingze
W68	ZL1YZ	Shanxi	Yingze
W69	PHYN	Yunnan	Kunming
W70	NS1NS	Shaanxi	Ningshan
W71	NS2NS	Shaanxi	Ningshan
W72	NS3NS	Shaanxi	Ningshan

The samples were obtained from network provider: W1-W33; The samples were obtained from experimental fields: W34-W72.
